# MALDI glycotyping of O-antigens from a single colony of gram-negative bacteria

**DOI:** 10.1038/s41598-024-62729-1

**Published:** 2024-06-03

**Authors:** Shogo Urakami, Hiroshi Hinou

**Affiliations:** 1 Laboratory of Advanced Chemical Biology, Graduate School of Life Science, Hokkaido University, Sapporo, 001-0021 Japan; 2 Frontier Research Center for Advanced Material and Life Science, Faculty of Advanced Life Science, Hokkaido University, Sapporo, 001-0021 Japan

**Keywords:** O-antigen, Biotyping, LPS, MALDI, Glycotyping, Single colony, Mass spectrometry, Polysaccharide sequencing, Bacterial techniques and applications, Diagnostic markers, Biotechnology, Industrial microbiology

## Abstract

Polypeptide-targeted MALDI-TOF MS for microbial species identification has revolutionized microbiology. However, no practical MALDI-TOF MS identification method for O-antigen polysaccharides, a major indicator for epidemiological classification within a species of gram-negative bacteria, is available. We describe a simple MALDI glycotyping method for O-antigens that simultaneously identifies the molecular mass of the repeating units and the monosaccharide composition of the O-antigen. We analyzed the *Escherichia coli* O1, O6, and O157-type strains. Conventional species identification based on polypeptide patterns and O-antigen polysaccharide typing can be performed in parallel from a single colony using our MALDI-TOF MS workflow. Moreover, subtyping within the same O-antigen and parallel colony-specific O-antigen determination from mixed strains, including the simultaneous identification of multiple strains-derived O-antigens within selected colony, were performed. In MALDI glycotyping of two *Enterobacteriaceae* strains, a *Citrobacter freundii* strain serologically cross-reactive with *E. coli* O157 gave a MALDI spectral pattern identical to *E. coli* O157. On the other hand, an *Edwardsiella tarda* strain with no reported O-antigen cross-reactivity gave a MALDI spectral pattern of unknown O-antigen repeating units. The method described in this study allows the parallel and rapid identification of microbial genera, species, and serotypes of surface polysaccharides using a single MALDI-TOF MS instrument.

## Introduction

**Figure 1:**
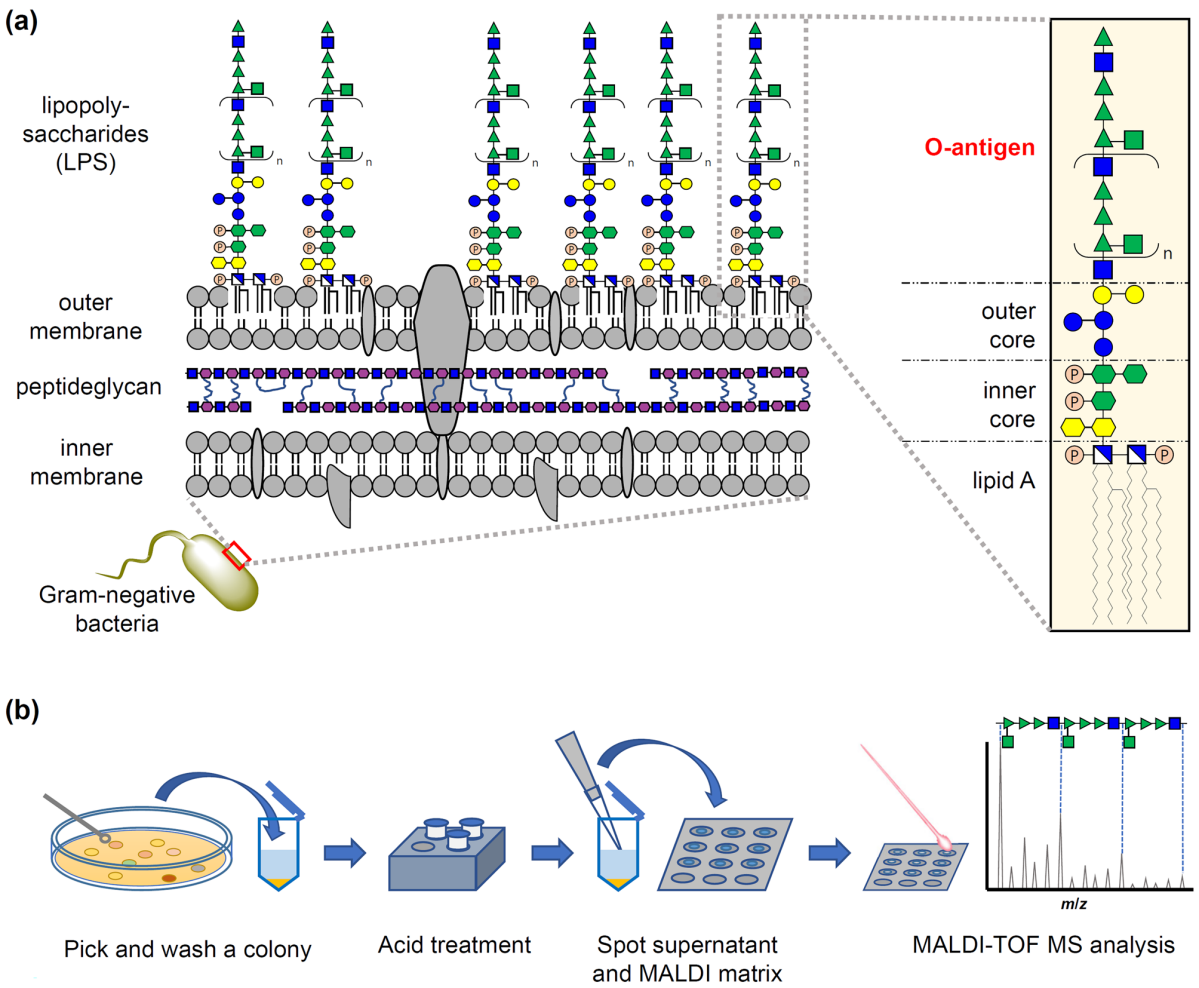
(**a**) Schematic cell membrane structure of gram-negative bacteria. (**b**) Workflow of MALDI glycotyping of O-antigens.

Matrix-assisted laser desorption/ionization time-of-flight mass spectrometry (MALDI-TOF MS)-based microbial species identification primarily targets the polypeptides comprising the ribosome. MALDI-TOF MS is the new standard in microbiology due to its simple method, high speed, and low cost^1^. However, the polypeptide-targeted MALDI typing method has limited accuracy for subtyping within the species, such as serotyping lipopolysaccharide (LPS) O-antigens^2,3^. The LPS structure is classified according to the glycolipid moieties into lipid A, core oligosaccharides, and outmost O-antigen polysaccharides^4^ (Fig. 1a). The O-antigen is a fundamental indicator for intraspecies subtyping of pathogenic gram-negative bacteria, such as O157 of enterohemorrhagic *Escherichia coli*^4,5^ due to its cell surface coverage and structural diversity. Coliform bacteria are the most abundant microorganisms in the intestinal flora of mammals and birds and the major pathogen involved in foodborne illness. The O-antigens of coliform bacteria are their primary epidemiological indicator^6,7^. Currently, *E. coli* has 181 reported O-antigens, and their classification numbers have become names in the taxonomy and epidemiology of the strains and are widely used for subtyping other gram-negative bacteria^7,8^. Determining the O-antigen glycan structure requires much time, money, and sophisticated techniques^9^; thus, indirect methods using probe molecules, such as selective mediums, antibodies, and polymerase chain reaction (PCR) primers, have been used for a long time to classify O-antigens. However, this probe-molecule-based classification is time-consuming and costly to cover the high diversity and variability of O-antigens. The glycan structures differ even within types diagnosed as the same O-antigen using probe-based methods (e.g., O1A-C)^7^. Therefore, non-probe identification methods for O-antigen glycan structures would expand the application of microbial identification, including for epidemiological subtyping. Although examples of O-antigen analysis using MALDI-TOF MS with isolated LPS^10–13^ and engineered glycoprotein vaccines^14^ exist, a rapid O-antigen identification method from a single bacterial colony or a polypeptide-based species identification method has not been reported. We recently developed a MALDI-TOF MS matrix for glycan-selective ionization and analysis of glycoconjugates^15^, called MALDI glycotyping, which can be applied for direct analysis of glycan patterns in glycoproteins and biofluids, such as egg whites^16,17^. Here, we report a simple and practical workflow for MALDI glycotyping of O-antigens from a single colony of gram-negative bacteria within 1 h (Fig. 1b). To optimize and validate this identification workflow, we selected four *E. coli* strains (Table 1), including two O157 strains and two strains with unidentified subtypes within the O-antigens O1 and O6.  We also report MALDI glycotyping of O-antigens of two *Enterobacteriaceae *strains.

**Table 1:** Basic information on the *E. coli* strains and potential O-antigen structures used in this study.

Strain (O-antigen)	O-antigen subtype	Unit sequence	Unit symbol	Unit exact mass
ATCC43888 (O157) ATCC700728 (O157)	–	→ 2)-d-Rha4NAc-(α1 → 3)-l-Fuc-(α1 → 4)-d-Glc-(β1 → 3)-d-GalNAc-(α1 →		698.27
ATCC11775 (O1)	O1A^20,21^	→ 3)-[d-ManNAc-(β1 → 2)]-l-Rha-(α1 → 3)-l-Rha-(α1 → 3)-l-Rha-(β1 → 4)-d-GlcNAc-(β1 →		844.33
	O1B^22^	→ 3)-[d-ManNAc-(β1 → 2)]-l-Rha-(α1 → 2)-l-Rha-(α1 → 2)-d-Gal-(α1 → 3)-d-GlcNAc-(β1 →		860.33
	O1C^22^	→ 3)-[d-ManNAc-(β1 → 2)]-l-Rha-(α1 → 2)-l-Rha-(α1 → 3)-d-Gal-(α1 → 3)-d-GlcNAc-(β1 →		860.33
ATCC25922 (O6)	Jansson^23^	→ 4)-d-GalNAc-(α1 → 3)-[d-Glc-(β1 → 2)]-d-Man-(β1 → 4)-d-Man-(β1 → 3)-d-GlcNAc-(α1 →		892.32
	Jann^24^	→ 4)-d-GalNAc-(α1 → 3)-[d-GlcNAc-(β1 → 2)]-d-Man-(β1 → 4)-d-Man-(β1 → 3)-d-GlcNAc-(α1 →		933.34

Potential subtypes within each O-antigen, reported repeating unit sequences of each O-antigen, glycan symbols of each repeating unit, and exact mass of the repeating units. D-Rha4NAc: 4-acetamido-4,6-dideoxy-d-mannose, L-Fuc: l-fucose (6-deoxy-l-galactose), D-Glc: d-glucose, D-GalNAc: 2-acetamido-2-deoxy-d-galactose, D-GlcNAc: 2-acetamido-2-deoxy-d-glucose, D-Man: d-mannose, D-ManNAc: 2-acetamido-2-deoxy-d-mannose, L-Rha: l-rhamnose (6-deoxy-l-mannose), D-Gal: d-galactose.

## Results

### Optimization and verification of MALDI glycotyping of O-antigens from single strains

**Figure 2:**
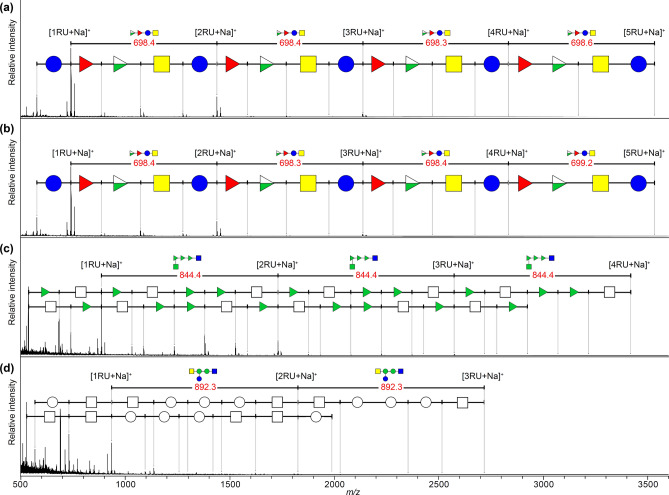
MALDI glycotyping spectrum of O-antigens from four *E. coli* strains: (**a**) ATCC43888; (**b**) ATCC700728; (**c**) ATCC11775; and (**d**) ATCC25922. RU: repeating unit; open white circle: hexose; open white square: N-acetylhexosamine. See Table 1 for other colored symbols indicating the monosaccharide components of the O-antigens.

Conventional methods for determining O-antigen structure require a large-scale culture of a single bacterial strain, LPS extraction, prolonged weak acid treatment for cleavage between lipid A and polysaccharide components, and a multi-step isolation and purification process^18^. We hypothesized that our glycan-selective ionization technique^16,17^ could identify the O-antigen by rapidly sorting the bacterial cell body as the solid-phase component and the O-antigen as the water-soluble component. First, we verified this workflow using O157-type *E. coli* (ATCC43888 strain) transferred from the agar medium into a microtube. After washing by simple suspension in water followed by centrifugation, 30 μL of water suspension (optical density [OD] 1.0–1.1) of bacterial cells were incubated with 100 mM hydrochloric acid suspension for 10 min at 90 °C in a PCR tube. Subsequently, the reaction suspension was centrifuged, and the supernatant was spotted onto a MALDI target plate. After drying the spotted samples, a mixture of 1,5-diaminonaphthalene (DAN)/2,5-dihydroxybenzoic acid (DHB)/sodium bicarbonate (Na) (2:10:1 [mol/mol/mol]) in 50% acetonitrile was added to the samples as a matrix for glycan selective ionization^17^. The subsequent MALDI-TOF MS analysis showed a characteristic peak pattern with Δ*m*/*z* 698 repeat, indicating the tetrasaccharide unit of the O157 antigen (typical spectra after optimization are shown in Fig. 2a, original spectra of this trial are provided in Supplementary Fig. 1). The detailed analysis of the spectral patterns observed between tetrasaccharide repeating units 1 and 2 revealed the peak patterns of its monosaccharide unit corresponding to l-fucose (Δ*m*/*z* 146), 4-deoxy-4-acetamide-d-rhamnose (Δ*m*/*z* 187), 2-deoxy-2-acetamide-d-glucose (Δ*m*/*z* 203), and d-glucose (Δ*m*/*z* 162), respectively (Fig. 2a, Supplementary Fig. 1, Supplementary Table 1). The minimum volume of the bacterial cell suspension required for this O-antigen glycotyping workflow was 1.5 μL (Supplementary Fig. 2). The optimal concentration of HCl for O-antigen hydrolysis from the cell body was 50–100 mM (Supplementary Fig. 3), with 100 mM being the optimal concentration. The minimum density of the bacterial cell suspension for detecting the signal from the O157 antigen was OD 0.064 (Supplementary Fig. 4a). Moreover, higher OD values result in better MALDI glycotyping spectra of the O-antigen repeating units (Supplementary Fig. 4a). When a 2-mm-diameter single colony was suspended in 30 μL of water, the OD was approximately 0.4, indicating that a single colony of bacterial cells suspended in 1.5 µL of water before HCl treatment is the appropriate amount of bacteria for this workflow (Supplementary Fig. 4b,c). The O-antigen analysis of ATCC700728 showed a MALDI spectral pattern indicating the presence of the O157 antigen, similar to that of ATCC43888 (Fig. 2b, Supplementary Table 2).

The workflow was then used to analyze the O-antigen of the strain ATCC11775, which has the O1 antigen, first discovered in *E. coli* and detected in various pathogenic *E. coli*, including avian pathogenic *E. coli* (APEC)^19^. To date, three different O1-type *E. coli* subtypes have been reported: O1A-C (Table 1)^20–22^; however, the O1-subtype of ATCC11775 is unknown. The MALDI glycotyping spectrum of strain ATCC11775 showed that this strain has an O1A antigen with a repeating unit of Δ*m*/*z* 844, comprised of three deoxyhexoses (Δ*m*/*z* 146) and two N-acetylhexosamines (Δ*m*/*z* 203) (Fig. 2c, Supplementary Table 3). Two different glycan structures of the O6 antigen of *E. coli* were reported by Jansson et al.^23^ and Jann et al.^24^. We identified that strain ATCC25922 has a glycan unit with the O-antigen reported by Jansson et al.^23^ consisting of a repeating structure at Δ*m*/*z* 892 (Fig. 2d, Supplementary Table 4). The spectral pattern between the unit structures showed that this O-antigen unit comprised three hexoses (Δ*m*/*z* 162) and two N-acetylhexosamines (Δ*m*/*z* 203).

### Parallel analysis of polypeptide and O-antigen patterns from a single *E. coli* colony

**Figure 3:**
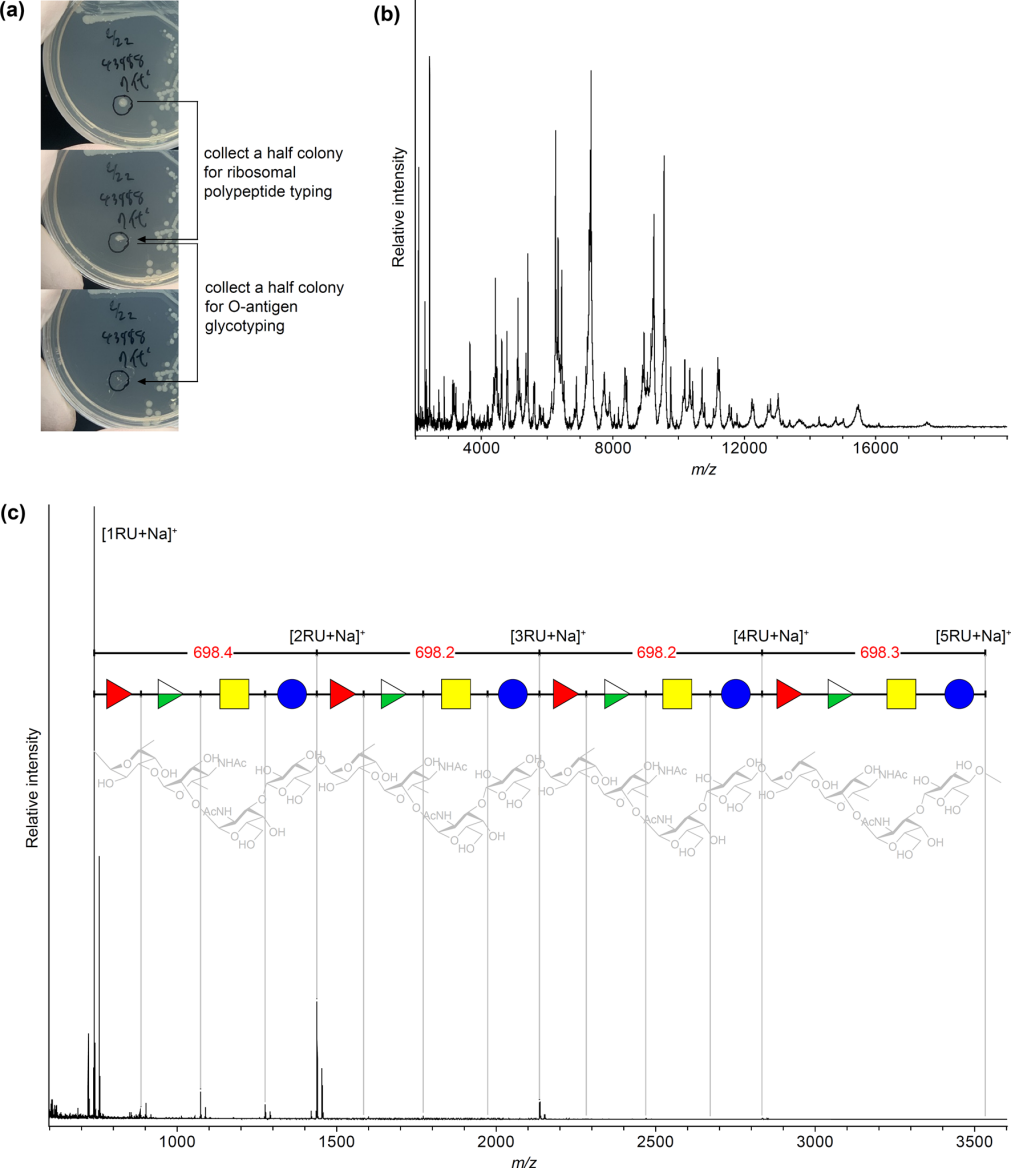
Parallel analysis of polypeptide and O-antigen patterns from a single colony of *E. coli* ATCC43888 strain: (**a**) two-step collection process from a single colony; (**b**) polypeptide-selective MALDI-TOF MS spectra (linear positive mode) for species fingerprinting; and (**c)** glycan-selective MALDI-TOF MS spectra (reflectron positive mode) for O-antigen structure determination. The identified O157 polysaccharide repeating unit mass cycles, mass-assigned sequence symbols, and corresponding chemical structures are overlaid. See Table 1 for symbols indicating the monosaccharide components of the O157 polysaccharide.

Polypeptide pattern analyses of four *E. coli* strains were performed using the formic acid-acetonitrile extraction method^25^ and measured using an α-cyano-4-hydroxycinnamic acid (CHCA) matrix. Nearly identical MALDI-TOF MS spectral patterns were observed for each strain (Supplementary Fig. 5). Lipid A pattern analysis^26^ of the four strains showed a nearly identical peak pattern (Supplementary Fig. 6). These conserved molecular markers can be used for rapid species identification, and when performed in parallel with our O-antigen identification method, a MALDI-TOF MS workflow of "species identification" and "intraspecific taxonomic O-antigen identification" can be performed. One colony (ca. 2.5 mm in diameter) of *E. coli* (ATCC43888) was divided into two parts. MALDI-typing experiments targeting ribosomal polypeptides and O-antigen polysaccharides were performed in parallel (Fig. 3). Intracellular polypeptides were extracted from one-half of the colony using the formic acid-acetonitrile method^25,27^. The supernatant was analyzed using a CHCA matrix (Fig. 3b). The other half of the colony was treated according to the MALDI-glycotyping workflow, and the acid-hydrolyzed supernatant was analyzed using a DAN/DHB/Na matrix (Fig. 3c). As a result, a polypeptide pattern spectrum indicative of *E. coli* and another one indicative of the repeated polysaccharide of O157 were obtained in parallel.

### Parallel identification of O-antigens in multiple colonies from a mixed strain

**Figure 4:**
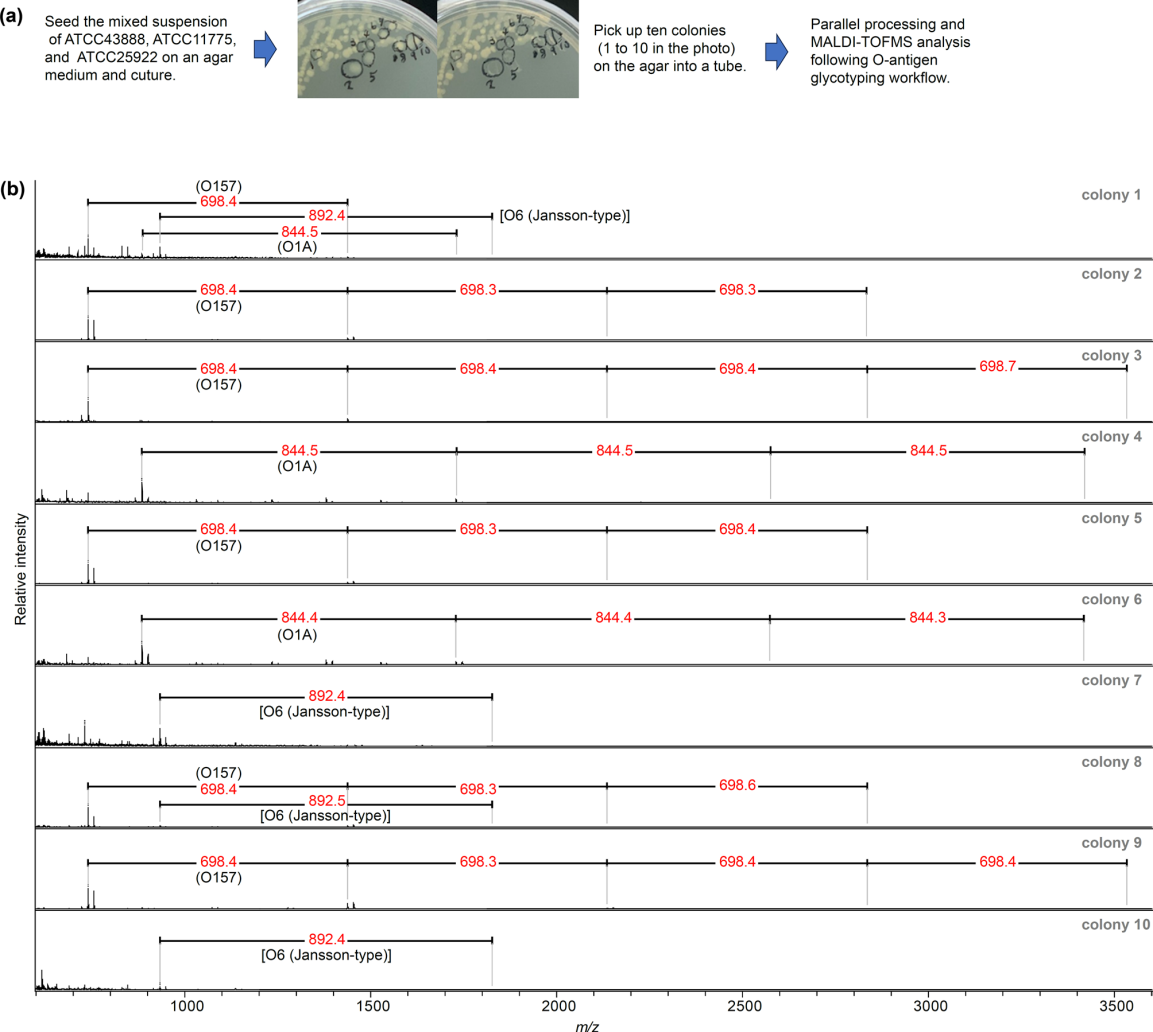
Parallel identification of the O-antigen of ten colonies from the *E. coli* strain mixture: (**a**) scheme of the culture and colony collection process and photographs taken before and after the colony collection and (**b**) O-antigen MALDI glycotyping spectra and identified O-antigen mass cycles for each of the ten colonies collected.

A mixture of three strains (ATCC11775, ATCC25922, and ATCC43888) was plated on agar medium, ten colonies were collected and placed into tubes, and MALDI glycotyping of their O-antigens was performed (Fig. 4). The glycotyping workflow of each of the 10 colonies was performed in parallel. The O-antigen pattern of each of them was successfully obtained. Eight of the ten selected colonies had a single O-antigen; colonies 4 and 6 were O1A; colonies 7 and 10 were O6 as the reported subtype by Jansson et al.^23^; and colonies 2, 3, 5, and 9 were O157. The MALDI-TOF MS spectra showed that colonies 1 and 8 were a mixture of multiple bacterial strains, with colony 1 being a mixture of all three strains and colony 8 being a mixture of the O6 and O157 type strains.

### MALDI glycotyping of O-antigens in *Enterobacteriaceae* strains

**Figure 5:**
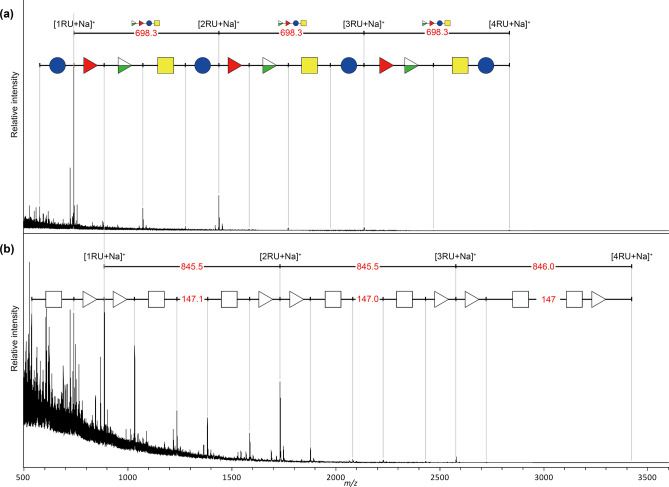
MALDI glycotyping spectrum of O-antigens for two *Enterobacteriaceae* strains; (**a**) *Citrobacter freundii* strain (NBRC 16624); and (**b**) *Edwardsiella tarda* strain (ATCC15947). RU: repeating unit; open white triangle: deoxy hexose; open white square: N-acetylhexosamine.

The family *Enterobacteriaceae* consists of facultative anaerobic gram-negative bacteria, and their O-antigens have acquired a high degree of diversity through horizontal interspecies transmission along with pathogenic factors. The family *Enterobacteriaceae* is a major factor in food-borne enteritis and zoonotic diseases; however, since they are mainly composed of resident bacteria, rapid identification of their O-antigen is a key epidemiological technology^28^. We measured the O-antigen of two non-*E. coli* *Enterobacteriaceae* species, *Citrobacter freundii* (NBRC 16624) and *Edwardsiella tarda* (ATCC15947), using MALDI glycotyping (Fig. 5). *C. freundii* (NBRC 16624) strain has identical O-antigen structure with *E. Coli* O157-type strains^29^. The MALDI-TOF MS spectra of *C. freundii* (NBRC 16624; Fig. 5a, Supplementary Table 5) obtained by the glycotyping workflow showed that the strain had an O-antigen strucuture identical to that of *E. coli* O157-type strains (ATCC43888 and ATCC700728; Fig. 2a and b, respectively). *Edwardsiella tarda* (ATCC15947) is a quality control strain of *E. tarda* with a structurally unknown O1483 serotype^30^. The MALDI glycotyping spectrum of strain ATCC15947 showed that the O-antigen of this strain had a repeating unit of Δ*m*/*z* 846, comprising two deoxyhexoses (Δ*m*/*z* 146), two N-acetylhexosamines (Δ*m*/*z* 203), and one unassignable Δ*m*/*z* 147 component (Fig. 5b, Supplementary Table 6).

## Discussion

We demonstrate a rapid MALDI glycotyping method for O-antigen identification using four *E. coli* strains and two non- *E. coli Enterobacteriaceae* strains. We used a combination of short heat treatment with HCl, a volatile strong acid, and a matrix that allows selective glycan ionization. By centrifuging, we separated the cell body of the microorganism as a solid-phase component with high specific gravity and the hydrolyzed O-antigen polysaccharides as the supernatant. We then placed the supernatant on a MALDI target plate. To avoid disturbing the selective glycan ionization, the volatile acid in the supernatant was removed by drying on the MALDI target plate, and the remaining O-antigen mixture was mixed with the matrix.

The O-antigen MALDI glycotyping workflow is characterized by rapidity, one-colony typing sensitivity, and simple operations that can be performed in parallel. This MALDI-TOF MS O-antigen identification method can be performed in parallel with polypeptide species identification methods, allowing species and intraspecies classifications such as "*E. coli*" + "O157" to be performed simultaneously without using probe molecules.

Several attempts have been reported to classify O-antigens based solely on the polypeptide pattern of ribosomes using MALDI-TOF MS. However, indirect prediction methods may make identifying glycan diversity within the same O-antigen or changes to new O-antigen glycan sequences difficult. Furthermore, the spectral patterns obtained by MALDI glycotyping for O-antigen identification reflected the O-antigen sequences. Therefore, unlike fingerprinting-type identification or genomic sequence-based analysis, this method can detect different O-antigen chemical structures that exhibit the same antigenic properties or unknown structures.

For the MALDI glycotyping of O-antigens, we chose random hydrolysis with a strong acid to obtain O-antigen spectral patterns comprised of free glycans, which was confirmed by the O-antigen analysis of the strain ATCC43888 using a glycoblotting method^31,32^. This method can capture the free glycans at their reducing end and label them with benzyloxyamine (BOA) with an increase in mass (Δ*m*/*z* 105) of the O157 sequence pattern (Supplementary Fig. 7). The monosaccharide composition data of the O-antigen unit structure observed in this random hydrolysis was confirmed in MALDI-TOF/TOF MS analysis of the periodic O-antigen unit precursor ion signal (Supplementary Figs. 8–10). Since the current MALDI-TOF MS instruments used for microbial species identification lack reflectron mode and TOF/TOF analysis capability^1,2^, an O-antigen identification workflow without TOF/TOF analysis is suitable for MALDI-TOF MS instruments available for clinical microbiology.

Although mass spectrometry has limitations in determining the detailed position/stereochemistry of unknown molecules, chemical structural identity can be demonstrated from the identity of fragment patterns. *Citrobacter freundii* NBRC 16624 strain that serologically shows cross-reactivity with *E. coli* O157 and the two *E. coli* O157 strains had identical MALDI-TOF MS spectral patterns, indicating that these three strains have the same O-antigen sequence. *Edwardsiella tarda* ATCC15947 strain has a MALDI-TOF MS spectral pattern indicating the presence of unknown O-antigen repeating units. The exact chemical structure of unknown O-antigens like this ATCC15947 strain can be determined by additional NMR and monosaccharide analyses^7,9^.The synthesis and external presentation of periodic polysaccharides, including exopolysaccharides of gram-positive bacteria and capsular polysaccharides, are common survival mechanisms in microorganisms^33,34^. The workflow described in this study can promote the epidemiology and industrial use of microbial polysaccharide phenotypes. In addition, this workflow allows the de novo sequencing of phenotypic O-antigens without the need for probes, which enables the discovery of "new" O-antigen pathotypes and their immediate diagnosis in remote areas by sharing information via the Internet. Research on the expansion of these applications is ongoing.

## Methods

### Materials

All *E. coli* strains, American Type Culture Collection (ATCC) 25922, ATCC43888, ATCC11775, and ATCC700728, and *E. tarda* strain (ATCC15947) were purchased from Microbiologics (St. Cloud, MN, USA). *C*. *freundii* strain (NBRC16624) was purchased from Biological Resource Center, National Institute of Technology and Evaluation (NBRC, Tokyo, Japan). The standard method agar ''DAIGO No. 802'' was obtained from Nihon Pharmaceutical Co., Ltd. (Osaka, Japan). Acetic acid, guanidine-HCl, ammonium bicarbonate, triethylamine, dioxan, DHB, sodium bicarbonate, acetonitrile (HPLC grade), HCl, and chloroform were purchased from Wako Pure Chemical Industries, Ltd. (Osaka, Japan). The 2,2,2-trifluoroacetic acid (TFA) was purchased from Watanabe Chemical Industry Co. Ltd. (Hiroshima, Japan). The DAN and CHCA were obtained from Sigma-Aldrich Corp. (St. Louis, MO, USA). The 3-methyl-1-*p*-tolyltriazene and benzyloxyamine hydrochloride were purchased from Tokyo Chemical Industry Co., Ltd. (Tokyo, Japan). Blot GlycoH beads were acquired from Sumitomo Bakelite Co., Ltd. (Tokyo, Japan). Acetic anhydride was purchased from Nacalai Tesque Inc. (Kyoto, Japan). All the water used in this study was freshly prepared using Milli-Q^®^ water (Direct-Q 3 UV; Merck Millipore, Tokyo, Japan).

### Matrices

For O-antigen analysis, 2 µL of 500 mM DHB in acetonitrile/water (9:1, v/v), 4 µL of 50 mM DAN in acetonitrile/water (1:1, v/v), and 1 µL of 100 mM NaHCO_3_ in water were mixed and diluted to 100 µL in an acetonitrile/water (1:1 v/v) solution. The DAN/DHB/Na matrix was used within 12 h. CHCA (10 mM) matrix solution in acetonitrile/water/TFA (50:50:0.1, v/v/v) was used for ribosomal polypeptide typing. A DHB (10 mg/mL) matrix solution in chloroform/methanol (9:1, v/v) was used for lipid A typing. For O-antigen analysis using glycoblotting method, 2 µL of 500 mM DHB in acetonitrile/water (9:1, v/v) and 1 µL of 100 mM NaHCO_3_ in water were mixed and diluted to 100 µL in an acetonitrile/water (1:1 v/v) solution.

### O-antigen typing of a single strain

All bacterial strains including *E. coli, E. tarda*, and *C. freundii* were grown on agar plates. Several bacterial colonies on the agar plate were transferred into a 1.5-mL tube. The cells were suspended in 1 mL of water, centrifuged at 10,000×*g* for 2 min, and the supernatant was discarded (Microcentrifuge 201324.1 Rev A; Thermo Fisher Scientific, Hampton, NH, USA). This water-washing procedure was performed twice; the bacteria were resuspended in 30 µL of water, and the cell suspension was transferred into PCR tubes. The OD of the suspension was monitored using a Photo Absorbance Meter (PAS-110-YU; Yamato Scientific Co., Ltd., Tokyo, Japan) and diluted to an OD of 1.7–1.8. From each suspension, 1.5 µL was transferred to another PCR tube and mixed with 0.5 µL of 400 mM HCl (total HCl concentration, 100 mM). The mixtures were incubated for 10 min at 90 °C and centrifuged at 20,000×*g* for 5 min (D3024 High-Speed Microcentrifuge; DLAB Scientific Co., Ltd., Beijing, China). The supernatant (0.35 µL) was loaded onto a MALDI target plate and dried at room temperature (20–25 ºC). The matrix solution (0.35 μL, DAN/DHB/Na) was deposited on the sample pre-spotted position and dried at room temperature.

### Ribosomal polypeptide typing (formic acid-acetonitrile extraction method)

Several colonies of bacterial cells grown on an agar culture plate were transferred into a 1.5-mL tube and suspended in 250 µL of water. Ethanol (1 mL) was added, and the mixture was resuspended and centrifuged at 10,000×*g* for 2 min. The supernatants were removed, and the pellets were dried. The pellets were mixed with 50 µL of 70% formic acid and then with acetonitrile (50 µL). The mixture was centrifuged at 15,000×*g* for 2 min. 0.35 µL of the supernatant was overlaid on 0.35 µL of the CHCA matrix pre-spotted target plate and dried at room temperature.

### Lipid A typing

The method up to the measurement of OD was the same as that for the O-antigen measurement. Each suspension (15 µL) was transferred into another PCR tube and suspended with 5 µL of 400 mM HCl (total HCl concentration of 100 mM). The mixture was incubated for 10 min at 90 °C and centrifuged at 20,000×*g* for 5 min. The supernatant was discarded, mixed with 100 µL of water, and centrifuged at 20,000×*g* for 5 min. The supernatant was discarded, and the remaining pellet was dried. The dried pellet was resuspended in 2 µL of water, and 0.2 µL of the suspension was loaded onto a MALDI target plate. The DHB matrix solution (0.60 μL) was deposited on the sample pre-spotted position and dried at room temperature.

### O-antigen typing from a single colony

A single colony of bacterial cells grown on an agar culture plate was picked, transferred into a 1.5 mL tube, suspended in 30 µL water, and centrifuged at 15,000×*g* for 2 min. The supernatant (28.5 µL) was removed, and 1.5 µL of the suspension of the remaining pellets was transferred into a PCR tube and mixed with 0.5 µL of 400 mM HCl (total HCl concentration of 100 mM). These mixtures were incubated for 10 min at 90 °C and centrifuged at 20,000×*g* for 5 min. 0.35 µL of the supernatant was loaded on a MALDI target plate and dried at room temperature. The matrix solution (0.35 μL, DAN/DHB/Na) was deposited on the sample pre-spotted target plate and dried at room temperature.

### O-antigen typing using the glycoblotting method

Treatment similar to the optimized O-antigen process was performed on the ATCC43888 strain. The strain was washed similarly to prepare a bacterial suspension with an OD of 1.9–2.0. Subsequently, 60 µL of the strain suspension was added to 20 µL of 400 mM HCl and incubated at 90 °C for 10 min. After incubation, the sample was centrifuged, and 60 µL of the supernatant was transferred to a 1.5 mL tube and dried in a SpeedVac (Thermo Fisher Scientific, Waltham, MA, USA). Blot GlycoH beads (250 µL, 10 mg/mL) were dispensed into a well of a 96-well multiScreen Solvert filter plate (Millipore, Billerica, MA, USA). The filter plate was then attached to a vacuum to remove water. The dried O-antigen sample released from ATCC43888 was dissolved in 60 µL of water, and all the solution was transferred to a well with 180 µL of 2% acetic acid in acetonitrile. The 96-well filter plate was incubated at 80 °C for 45 min. Then, the treated sample was washed with 200 µL of 2 M guanidine-HCl in 16.6 mM ammonium bicarbonate, water, and 1% triethylamine in methanol. Each washing process was performed twice, and a vacuum was performed after each step. The unreacted hydrazide functional groups on the beads were capped with acetyl groups by incubating with 100 µL of 2% acetic anhydride in methanol for 30 min at room temperature. The solution was then removed using a vacuum and washed twice with 200 µL of 10 mM HCl, methanol, and dioxane. On-bead methyl esterification was performed by adding 100 µL of 100 mM 3-methyl-1-*p*-tolyltriazene in dioxane into the sample well and incubating at 60 °C for 90 min. The plate was sequentially washed twice with 200 µL of dioxane, water, methanol, and water. The captured O-antigen-derived glycans on the BlotGlycoH beads were released and labeled with benzyloxyamine (BOA). The labeling and releasing process was performed by adding 20 µL of 50 mM BOA-HCl and 180 µL of 2% acetic acid in acetonitrile at 80 °C for 45 min. BOA-labeled glycans were eluted twice with 200 µL of water. The eluted solutions were mixed and dried using a SpeedVac. The dried sample was dissolved in 10 µL of water. The sample solution (0.35 µL) and DHB/Na matrix solution (0.35 µL) were loaded on a MALDI target plate and dried under air blow.

### Mass spectrometry

All spectra were acquired using an Ultraflex III instrument (Bruker, Bremen, Germany) equipped with a 200 Hz Smartbeam Nd:YAG laser (355 nm) and operated with flexControl (version 3.4, https://www.bruker.com/). O-antigen spectra were acquired in the positive reflectron mode with 1000 laser shots and random walks. Lipid A spectra were acquired in the negative reflectron mode with 1000 laser shots and a random walk. Ribosomal polypeptide spectra were acquired in positive linear mode with 1000 laser shots and a random walk. The generated ions accelerated to a kinetic energy of 25.0 kV. The low-mass-ion deflector cut-off was set at 700 Da. In the LIFT-TOF/TOF mode, the fragment ions were accelerated to 8 kV in the MALDI ion source and selected at a time gate. The selected ions were further accelerated to 19 kV in the LIFT cell. The metastable PSD ions were analyzed. The raw MALDI-TOF and MALDI-TOF/TOF MS data were annotated using the flexAnalysis (version 3.4, https://www.bruker.com/).

### Supplementary Information

Supplementary Information.

## Data Availability

Data is provided within the manuscript or supplementary information files.
