# Supplementary material for: MALDI glycotyping of O-antigens from a single colony of gram-negative bacteria
Source: Sci Rep. 2024 Jun 3;14:12719. doi: 10.1038/s41598-024-62729-1 (PMC11148006; doi:10.1038/s41598-024-62729-1)
Supplement: Supplementary file 1 — Supplementary Information. [file 41598_2024_62729_MOESM1_ESM.pdf]

supplementary information

MALDI glycotyping of O-antigens from a single colony of gram-negative bacteria

Shogo Urakami[1] and Hiroshi Hinou[1,2,*]

[1]Laboratory of Advanced Chemical Biology, Graduate School of Life Science, Hokkaido University, Sapporo 001-0021, Japan.

[2]Frontier Research Center for Advanced Material and Life Science, Faculty of Advanced Life Science, Hokkaido University, Sapporo 001-0021, Japan.

*Corresponding author: hinou@sci.hokudai.ac.jp

Supplementary Fig. 1~10.

Supplementary Table 1~6.

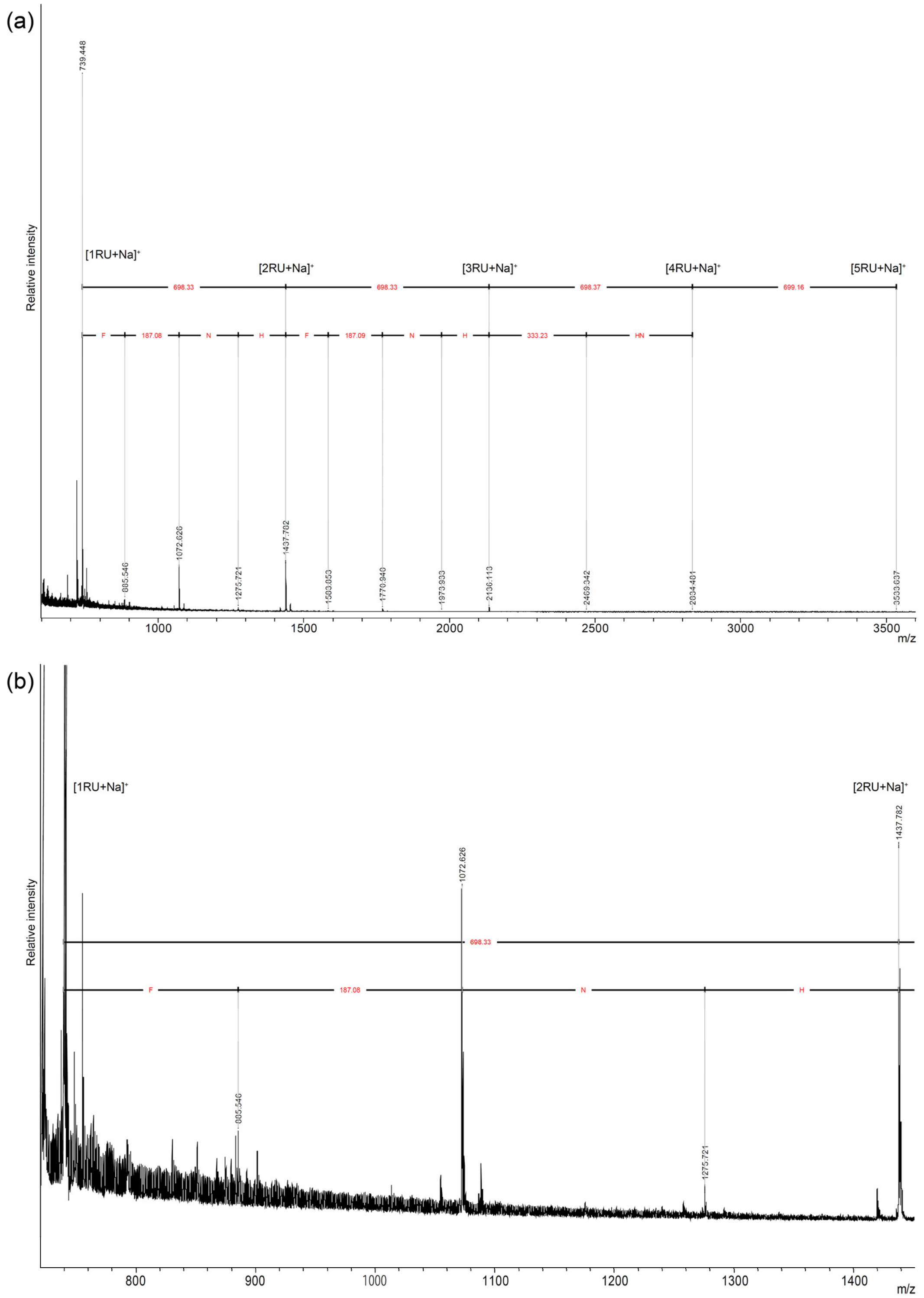

Supplementary Fig. 1 │ MALDI glycotyping spectrum of a *E. coli* (ATCC43888) suspension (30 µL, OD 1.060), treated with 100 mM HCl, at 90 °C for 10 min, the supernatant was analyzed with DAN/DHB/Na matrix: **a**, whole spectrum of O-antigen derived signals; **b**, signals between 1st repeating unit (RU) and 2nd RU.

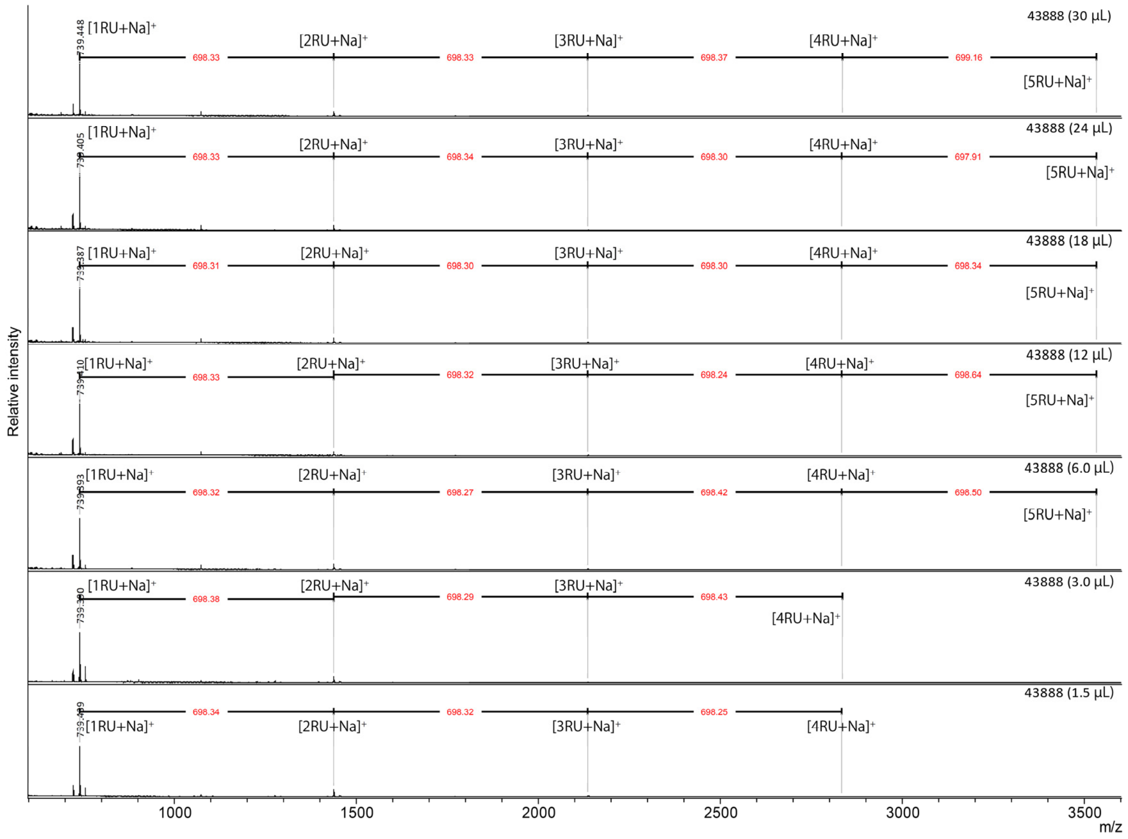

Supplementary Fig. 2 │ MALDI glycotyping spectrum from different volume of *E. coli* (ATCC43888) suspension (30 µL, 24 µL, 18 µL, 12 µL, 6.0 µL, 3.0 µL, 1.5 µL) of OD 1.060, treated with 100 mM HCl at 90 °C for 10 min, 0.35 µL of the supernatant was analyzed with DAN/DHB/Na matrix.

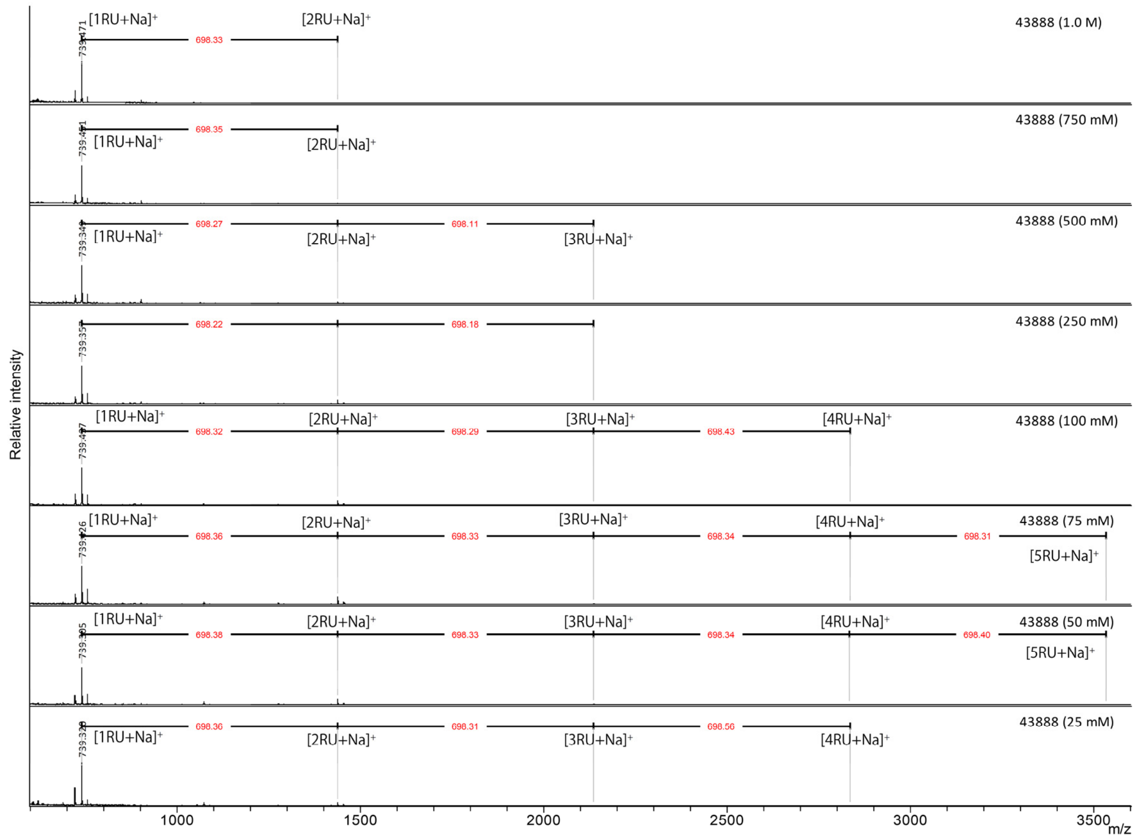

Supplementary Fig. 3 │ MALDI glycotyping spectrum of *E. coli* ATCC43888 suspension (1.5 µL, OD 1.024) treated with HCl at different final concentration (1000 mM, 750 mM, 500 mM, 250 mM, 100 mM, 75 mM, 50 mM, 25 mM) at 90 °C for 10 min, 0.35 µL of the supernatant was analyzed with DAN/DHB/Na matrix.

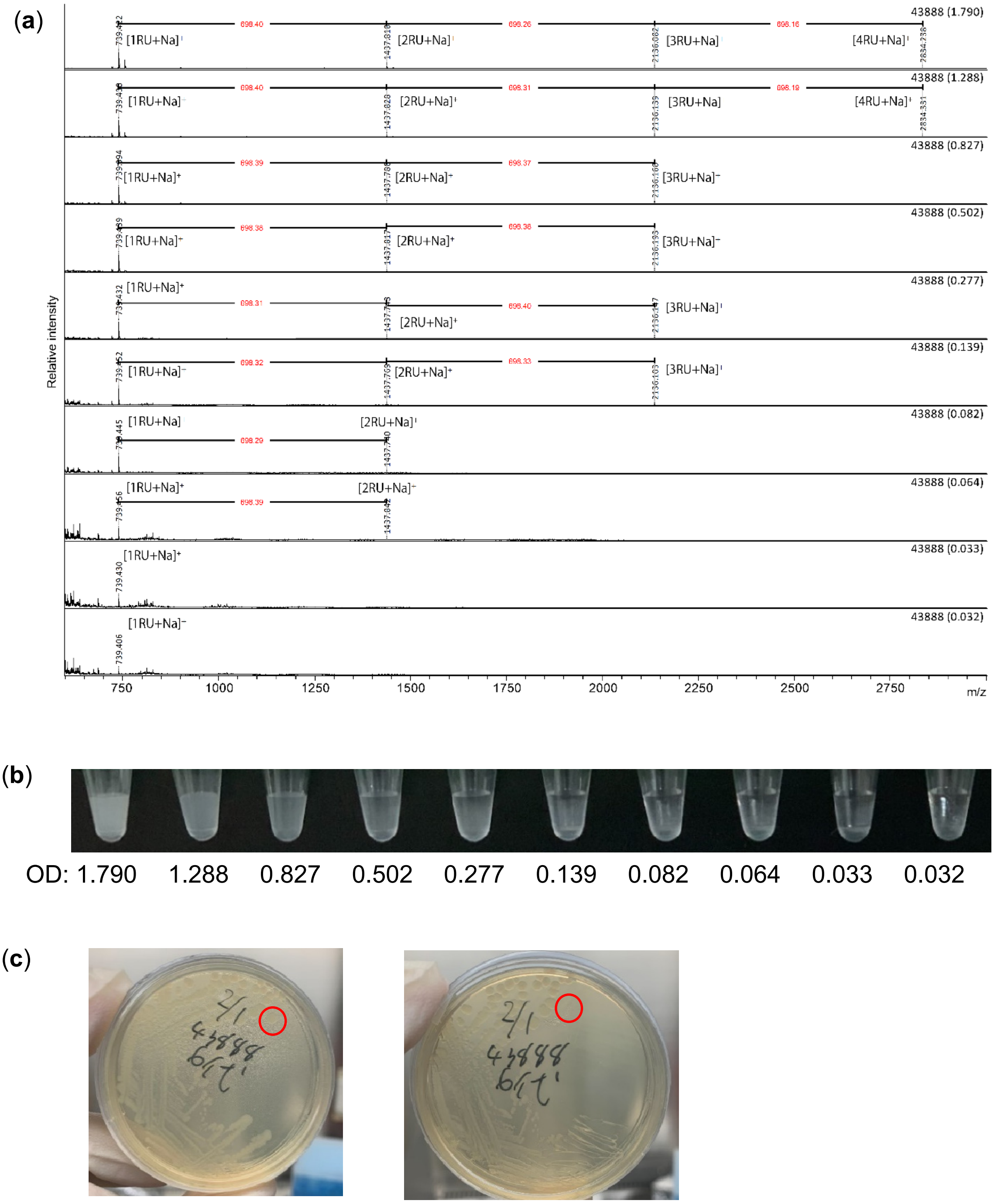

Supplementary Fig. 4 │ a, MALDI glycotyping spectrum from different OD (1.790, 1.288, 0.827, 0.502, 0.277, 0.139, 0.082, 0,064, 0.033, 0.032) of *E. coli* ATCC43888 suspension (1.5 μL), treated with 0.5 μL of 400 mM HCl at 90 °C for 10 min, 0.35 μL of the supernatant was analyzed with DAN/DHB/Na matrix. **b**, *E. coli* (ATCC43888, 30 μL in clear PCR tube) suspension of different optical density prepared by 2-fold dilution from OD 1.790 suspension. **c**, Before and after collect a single colony highlighted by red circle in ager plate to give 30 μL of OD 0.40 suspension in water.

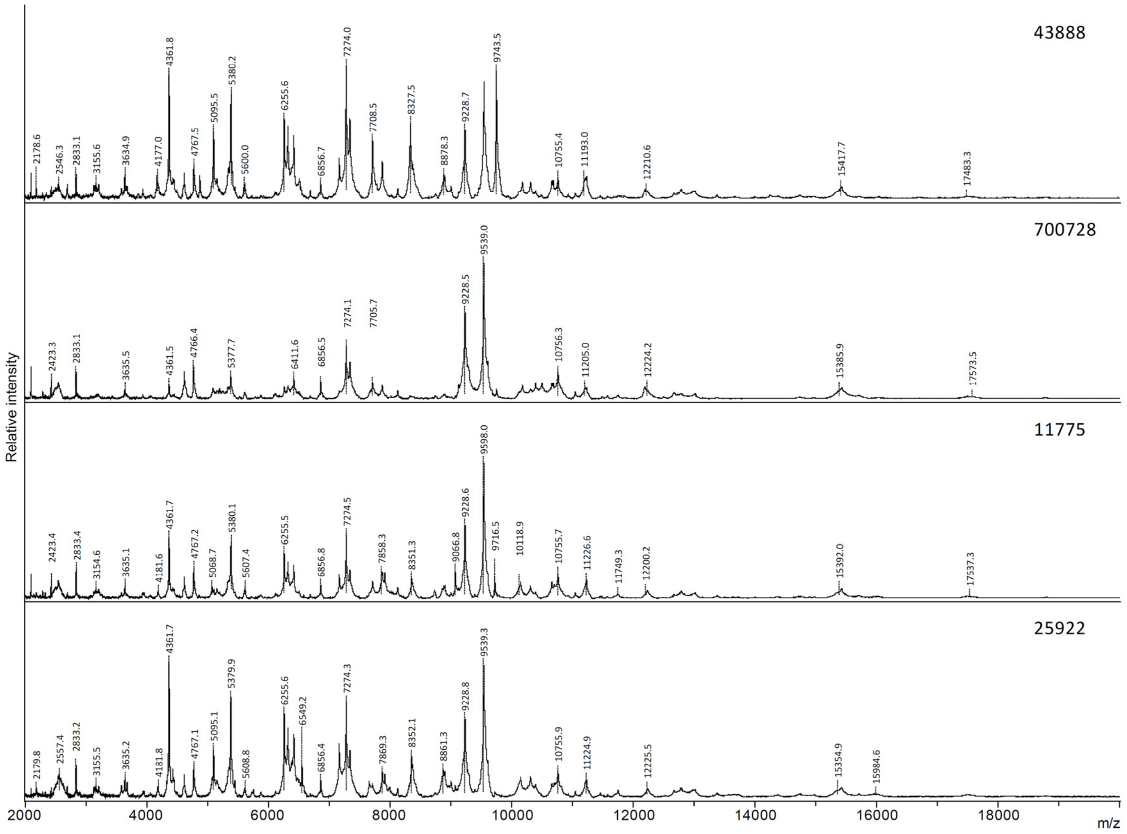

Supplementary Fig. 5 │ MALDI polypeptide typing spectrum of *E. coli* ATCC43888 (O157), ATCC700728 (O157), ATCC11775 (O1), and ATCC25922 (O6) obtained by formic acid-acetonitrile extraction method.

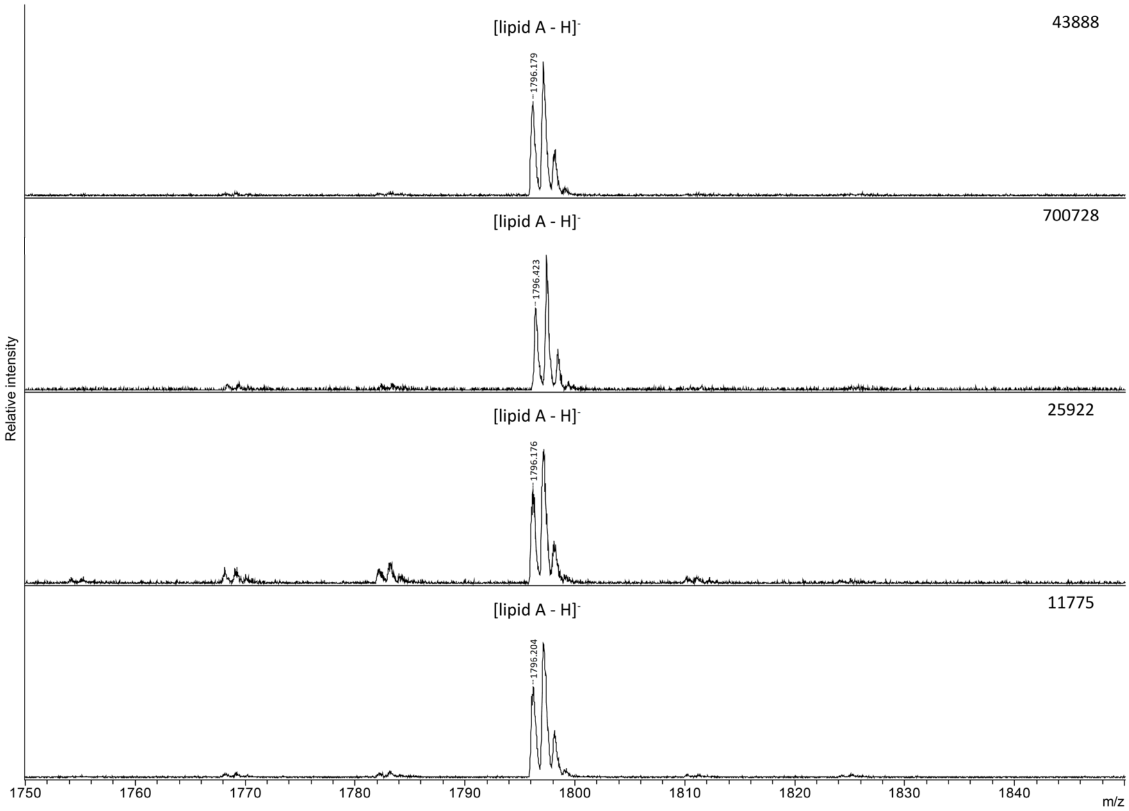

Supplementary Fig. 6 │ MALDI lipid A typing spectrum of *E. coli* ATCC43888 (O157), ATCC700728 (O157), ATCC11775 (O1), and ATCC25922 (O6) obtained by washed and HCl treated cell smear method.

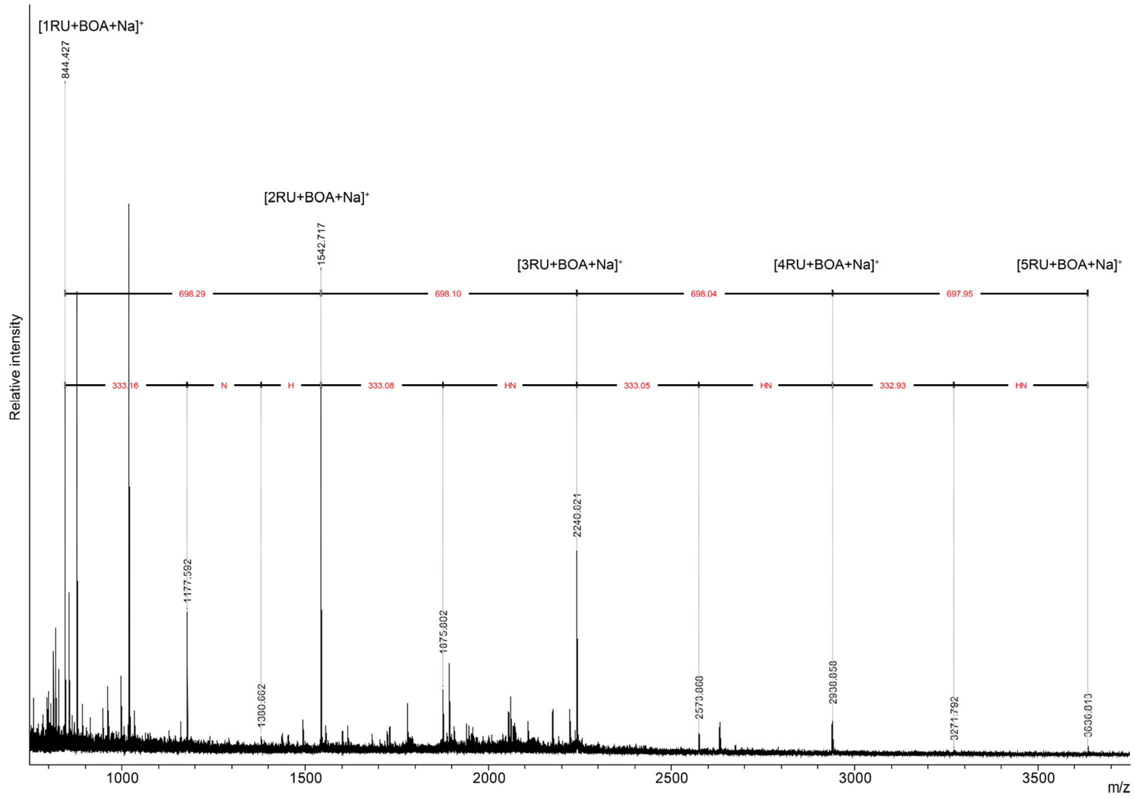

Supplementary Fig. 7 | MALDI spectrum of benzyloxyamine (BOA) labeled fragments of O157 polysaccharide of *E. coli* ATCC43888 captured by glycoblotting method from 100 mM HCl treated supernatant (obtained from 60 µL of OD 1.9-2.0 suspension of *E. coli* ATCC43888 cells). Δ*m*/*z* 105 shifted were observed according to the BOA labeling at reducing end of each glycan signals compared with the MALDI glycotyping peak signals in Fig. 2a.

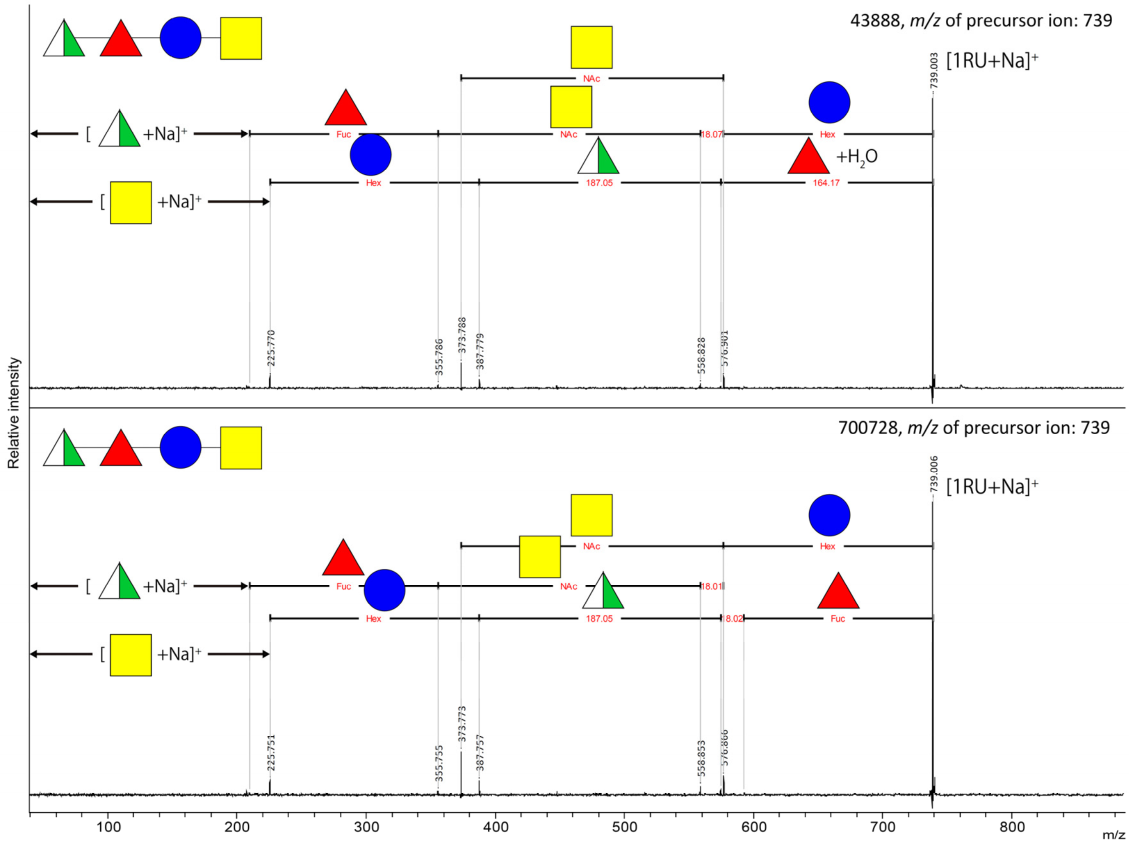

Supplementary Fig. 8 │ MALDI-TOF/TOF MS spectrum of a single repeating unit of O157-antigen derived precursor ions obtained from ATCC43888 (upper) and ATCC700728 (lower) strains.

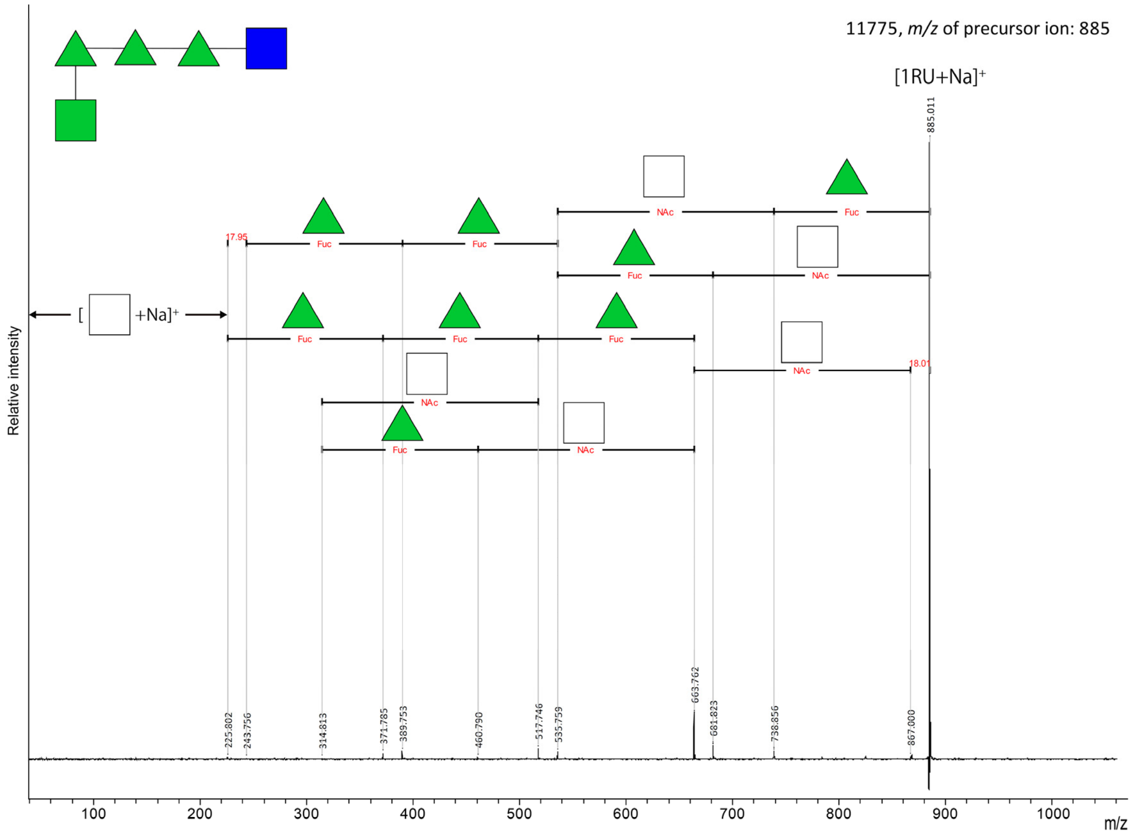

Supplementary Fig. 9 │ MALDI-TOF/TOF MS spectrum of a single repeating unit of O1A-antigen derived precursor ion obtained from ATCC11775 strain.

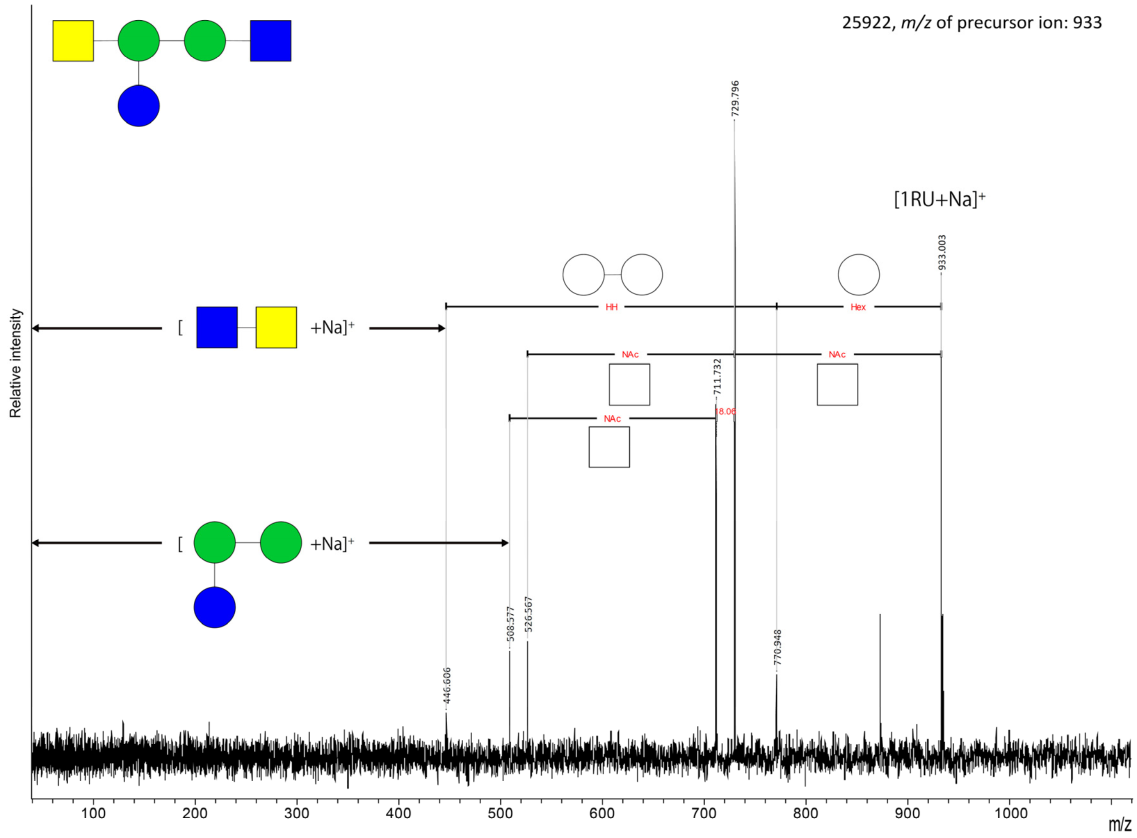

Supplementary Fig. 10 │ MALDI-TOF/TOF MS spectrum of a single repeating unit of O6-antigen (Jansson-type) derived precursor ion obtained from ATCC25922 strain.

Supplementary Table 1 │ List of products ion peaks of O157-antigen of ATCC43888 strain in Fig. 2a.

assigned structure	observed *m/z*	calculated *m/z*	*Δm/z*
[RhaNAc + Fuc + GalNAc + H_2O + Na]$^+$	577.344	577.222	0.122
[1RU + H_2O + Na]$^+$	739.461	739.275	0.186
[1RU + Fuc + H_2O + Na]$^+$	885.563	885.333	0.230
[1RU + RhaNAc + Fuc + H_2O + Na]$^+$	1072.666	1072.417	0.249
[1RU + RhaNAc + Fuc + GalNAc + H_2O + Na]$^+$	1275.763	1275.497	0.266
[2RU + H_2O + Na]$^+$	1437.836	1437.549	0.287
[2RU + Fuc + H_2O + Na]$^+$	1583.920	1583.607	0.312
[2RU + RhaNAc + Fuc + H_2O + Na]$^+$	1771.011	1770.692	0.319
[2RU + RhaNAc + Fuc + GalNAc + H_2O + Na]$^+$	1974.100	1973.771	0.329
[3RU + H_2O + Na]$^+$	2136.187	2135.824	0.363
[3RU + Fuc + H_2O + Na]$^+$	2282.251	2281.882	0.369
[3RU + RhaNAc + Fuc + H_2O + Na]$^+$	2469.305	2468.966	0.339
[3RU + RhaNAc + Fuc + GalNAc + H_2O + Na]$^+$	2672.501	2672.046	0.455
[4RU + H_2O + Na]$^+$	2834.500	2834.099	0.401
[4RU + RhaNAc + Fuc + H_2O + Na]$^+$	3167.405	3167.241	0.164
[5RU + H_2O + Na]$^+$	3533.116	3532.373	0.743

Supplementary Table 2 │ List of products ion peaks of O157-antigen of ATCC700728 strain in Fig. 2b.

assigned structure	observed *m/z*	calculated *m/z*	*Δm/z*
[RhaNAc + Fuc + GalNAc + H_2O + Na]$^+$	577.328	577.222	0.106
[1RU + H_2O + Na]$^+$	739.421	739.275	0.147
[1RU + Fuc + H_2O + Na]$^+$	885.534	885.333	0.201
[1RU + RhaNAc + Fuc + H_2O + Na]$^+$	1072.640	1072.417	0.223
[1RU + RhaNAc + Fuc + GalNAc + H_2O + Na]$^+$	1275.731	1275.497	0.234
[2RU + H_2O + Na]$^+$	1437.801	1437.549	0.251
[2RU + Fuc + H_2O + Na]$^+$	1583.903	1583.607	0.296
[2RU + RhaNAc + Fuc + H_2O + Na]$^+$	1770.986	1770.692	0.295
[2RU + RhaNAc + Fuc + GalNAc + H_2O + Na]$^+$	1974.072	1973.771	0.301
[3RU + H_2O + Na]$^+$	2136.141	2135.824	0.316
[3RU + Fuc + H_2O + Na]$^+$	2282.155	2281.882	0.273
[3RU + RhaNAc + Fuc + H_2O + Na]$^+$	2469.368	2468.966	0.402
[3RU + RhaNAc + Fuc + GalNAc + H_2O + Na]$^+$	2672.435	2672.046	0.389
[4RU + H_2O + Na]$^+$	2834.501	2834.099	0.403
[5RU + H_2O + Na]$^+$	3533.682	3532.373	1.309

Supplementary Table 3 │ List of products ion peaks of O1A-antigen of ATCC11775 strain in Fig. 2c.

assigned structure	observed *m/z*	calculated *m/z*	*Δm/z*
$[2Rha + NAc + H_2O + Na]^+$	536.278	536.196	0.082
$[3Rha + NAc + H_2O + Na]^+$	682.396	682.253	0.143
$[2Rha + 2NAc + H_2O + Na]^+$	739.446	739.275	0.171
$[1RU + H_2O +Na]^+$	885.518	885.333	0.185
$[1RU + Rha + H_2O +Na]^+$	1031.623	1031.391	0.233
$[1RU + NAc + H_2O +Na]^+$	1088.648	1088.412	0.235
$[1RU + 2Rha + H_2O +Na]^+$	1177.707	1177.449	0.258
$[1RU + Rha + NAc + H_2O + Na]^+$	1234.714	1234.470	0.244
$[1RU + 3Rha + H_2O + Na]^+$	1323.798	1323.507	0.291
$[1RU + 2Rha + NAc + H_2O + Na]^+$	1380.796	1380.528	0.268
$[1RU + 3Rha + NAc + H_2O + Na]^+$	1526.865	1526.586	0.279
$[1RU + 2Rha + 2NAc + H_2O + Na]^+$	1583.899	1583.607	0.291
$[2RU + H_2O + Na]^+$	1729.955	1729.665	0.289
$[2RU + Rha + H_2O + Na]^+$	1876.025	1875.723	0.302
$[2RU + NAc + H_2O + Na]^+$	1933.025	1932.745	0.280
$[2RU + 2Rha + H_2O + Na]^+$	2022.079	2021.781	0.297
$[2RU + Rha + NAc + H_2O + Na]^+$	2079.123	2078.803	0.321
$[2RU + 2Rha + NAc + H_2O + Na]^+$	2225.203	2224.861	0.342
$[2RU + 3Rha + NAc + H_2O + Na]^+$	2371.262	2370.918	0.343
$[2RU + 2Fuc + 2NAc + H_2O + Na]^+$	2428.260	2427.940	0.320
$[3RU + H_2O + Na]^+$	2574.375	2573.998	0.377
$[3RU + Rha + H_2O + Na]^+$	2720.510	2720.056	0.454
$[3RU + NAc + H_2O + Na]^+$	2777.460	2777.077	0.383
$[3RU + Rha + NAc + H_2O + Na]^+$	2923.502	2923.135	0.367
$[3RU + 2Rha + NAc + H_2O + Na]^+$	3069.526	3069.193	0.333
$[3RU + 3Rha + NAc + H_2O + Na]^+$	3215.542	3215.251	0.291
$[4RU + H_2O + Na]^+$	3418.721	3418.330	0.391

Supplementary Table 4 │ List of products ion peaks of O6-antigen (Jansson-type) of ATCC25922 strain in Fig. 2d.

assigned structure	observed *m/z*	calculated *m/z*	*Δm/z*
$[3Hex + H_2O + Na]^+$	527.202	527.159	0.043
$[2Hex + NAc + H_2O + Na]^+$	568.268	568.185	0.083
$[3Hex + NAc + H_2O + Na]^+$	730.388	730.238	0.150
$[2Hex + 2NAc + H_2O + Na]^+$	771.431	771.265	0.167
$[1RU + H_2O + Na]^+$	933.520	933.318	0.202
$[1RU + Hex + H_2O + Na]^+$	1095.599	1095.370	0.229
$[1RU + NAc + H_2O + Na]^+$	1136.622	1136.397	0.225
$[1RU + 2Hex + H_2O + Na]^+$	1257.676	1257.423	0.253
$[1RU + Hex + NAc + H_2O + Na]^+$	1298.720	1298.450	0.270
$[1RU + 2NAc + H_2O + Na]^+$	1339.727	1339.476	0.251
$[1RU + 3Hex + H_2O + Na]^+$	1419.733	1419.476	0.257
$[1RU + 2Hex + NAc + H_2O + Na]^+$	1460.761	1460.503	0.258
$[1RU + Hex + 2NAc + H_2O + Na]^+$	1501.739	1501.529	0.210
$[1RU + 3Hex + NAc + H_2O + Na]^+$	1622.817	1622.555	0.262
$[2RU + H_2O + Na]^+$	1825.850	1825.635	0.215
$[2RU + Hex + H_2O + Na]^+$	1987.954	1987.688	0.266
$[2RU + NAc + H_2O + Na]^+$	2028.942	2028.714	0.228
$[2RU + 2Hex + NAc + H_2O +Na]^+$	2352.896	2352.820	0.076
$[2RU+3Hex+Nac+ H_2O +Na]^+$	2514.988	2514.873	0.116
$[3RU + H_2O + Na]^+$	2718.132	2717.952	0.180

Supplementary Table 5 │ List of products ion peaks of O-antigen of *Citrobacter freundii* (NBRC 16624) strain in Fig. 5a.

assigned structure	observed *m/z*	calculated *m/z*	*Δm/z*
[RhaNAc + Fuc + GalNAc + H_2O + Na] $^{+}$	577.282	577.22208	0.060
[1RU + H_2O + Na] $^{+}$	739.371	739.27491	0.096
[1RU + Fuc + H_2O + Na] $^{+}$	885.460	885.33282	0.127
[1RU + RhaNAc + Fuc + H_2O + Na] $^{+}$	1072.538	1072.41728	0.121
[1RU + RhaNAc + Fuc + GalNAc + H_2O + Na] $^{+}$	1275.639	1275.49666	0.143
[2RU + H_2O + Na] $^{+}$	1437.672	1437.54949	0.123
[2RU + Fuc + H_2O + Na] $^{+}$	1583.742	1583.6074	0.134
[2RU + RhaNAc + Fuc + H_2O + Na] $^{+}$	1770.822	1770.69186	0.130
[2RU + RhaNAc + Fuc + GalNAc + H_2O + Na] $^{+}$	1973.867	1973.77124	0.096
[3RU + H_2O + Na] $^{+}$	2135.937	2135.82407	0.113
[3RU + RhaNAc + Fuc + H_2O + Na] $^{+}$	2469.016	2468.96644	0.049
[4RU + H_2O + Na] $^{+}$	2834.235	2834.09865	0.137

Supplementary Table 6 │ List of products ion peaks of O-antigen of *Edwardsiella tarda* (ATCC15947) strain in Fig. 5b.

assigned structure	observed *m/z*	calculated *m/z*	*Δm/z*
[dHex + 1HexNAc + 147 + H_2O + Na] $^+$	537.353		
[dHex + 2HexNAc + 147 + H_2O + Na] $^+$	740.464		
[1RU + H_2O + Na] $^+$	886.546		
[1RU + dHex + H_2O + Na] $^+$	1032.640		
[1RU + dHex + HexNAc + H_2O + Na] $^+$	1235.785		
[1RU + dHex + HexNAc + 147 + H_2O + Na] $^+$	1382.877		
[1RU + dHex + 2HexNAc + 147 + H_2O + Na] $^+$	1585.989		
[2RU + H_2O + Na] $^+$	1732.079		
[1RU + dHex + H_2O + Na] $^+$	1878.178		
[1RU + dHex + HexNAc + H_2O + Na] $^+$	2081.253		
[1RU + dHex + HexNAc + 147 + H_2O + Na] $^+$	2228.302		
[1RU + dHex + 2HexNAc + 147 + H_2O + Na] $^+$	2431.387		
[3RU + H_2O + Na] $^+$	2577.593		
[3RU + dHex + H_2O + Na] $^+$	2723.726		
[4RU + H_2O + Na] $^+$	3423.554		
